# Species-specific renal and liver responses during infection with food-borne trematodes *Opisthorchis felineus*, *Opisthorchis viverrini*, or *Clonorchis sinensis*

**DOI:** 10.1371/journal.pone.0311481

**Published:** 2024-12-05

**Authors:** Oxana G. Zaparina, Yaroslav K. Kapushchak, Ekaterina A. Lishai, Sung-Jong Hong, Banchob Sripa, Maria Y. Pakharukova

**Affiliations:** 1 Institute of Cytology and Genetics, Siberian Branch of Russian Academy of Sciences (ICG SB RAS), Novosibirsk, Russia; 2 Department of Natural Sciences, Novosibirsk State University, Novosibirsk, Russia; 3 Center for Infectious Diseases and Vectors, Incheon National University, Incheon, Korea; 4 Faculty of Medicine, Department of Pathology, WHO Collaborating Centre for Research and Control of Opisthorchiasis (Southeast Asian Liver Fluke Disease), Tropical Disease Research Center, Khon Kaen University, Khon Kaen, Thailand; Niigata University of Pharmacy and Medical and Life Sciences, JAPAN

## Abstract

Three food-borne trematodes—*Opisthorchis felineus*, *Opisthorchis viverrini*, and *Clonorchis sinensis*—are closely related epidemiologically important species. Despite the similarity of their life cycles, these liver flukes also have marked differences in the geographical range, helminth biology, and hepatobiliary disorders. *O*. *viverrini* and *C*. *sinensi*s are classified as Group 1 biological carcinogens while *O*. *felineus* is not. Direct comparisons of systemic response to the liver fluke infections are unexplored aspects. This study was carried out to identify species-specific liver and kidney responses in the hamster models after the infection with one of the three liver flukes. Liver periductal-fibrosis development was similar between hamsters infected with *O*. *felineus* or *C*. *sinensis*, whereas biliary intraepithelial neoplasia development was noticed predominantly in *O*. *viverrini*–infected ones. Species-specific renal damage was detected, including progression of interstitial fibrosis and IgA deposition in glomeruli of *O*. *felineus*–infected hamsters and *C*. *sinensis*–infected ones. A strong correlation (R = 0.63; P = 0.0001) was found between periductal fibrosis in the liver and kidney interstitial fibrosis. Future comparative studies are needed to elucidate the development of serious complications during the long term of the infection, as well as under the influence of additional factors, including concomitant infections and the use of dimethylnitrosamine to clarify the mechanisms underlying the liver fluke-associated carcinogenesis. Thus, our findings may stimulate new comparative studies on the pathogenicity.

## 1. Introduction

Infection with foodborne trematodes is a serious public-health problem. Three species of liver flukes—*Opisthorchis viverrini* (Poirier, 1886), *Opisthorchis felineus* (Rivolta, 1884), and *Clonorchis sinensis* (Loos, 1907)—belong to the family Opisthorchiidae (Trematoda, Platyhelminthes, Digenea) and rank 8th in the global list of 24 clinically significant foodborne parasites [1].

These three liver flukes are closely related species sharing similar life cycle involving two intermediate hosts and one definitive host. Their final hosts are fish-eating mammals and human. Infection occurs after eating raw or undercooked freshwater fish of the Cyprinidae family [2]. Helminthic liver infections caused by invasion by *O*. *felineus*, *C*. *sinensis*, and *O*. *viverrini* have similar clinical manifestations, and are treated with same anthelmintic, praziquantel. These information may give impression that there is no difference among these three liver fluke infections.

Nonetheless, there are differences, primarily in the genetic material of these species, in particular, *O*. *felineus* and *C*. *sinensis* have seven pairs of chromosomes (2n = 14), while *O*. *viverrini* has six pairs (2n = 12) [3]. Geographic ranges of *O*. *felineus*, *O*. *viverrini*, and *C*. *sinensis* are very different and do not overlap (S1 Fig). For instance, *O*. *viverrini* has about 12 million infected people in Southeast Asia (Thailand, Lao PDR, Cambodia and Vietnam) [4]; *O*. *felineus* 1.2 million infected people in endemic regions of Russia and European countries [5]; and *C*. *sinensis* has infected 15–20 million people in China and South Korea [6,7]. At the same time, about 680 million people in the world are at risk of infection by these liver flukes [6]. Carcinogenic potentials are also different among these three species. *O*. *viverrini* and *C*. *sinensis* are recognized as group 1 biological carcinogens of cholangiocarcinoma in endemic areas [8].

In addition, liver fluke infections may be accompanied by the development of clinical symptoms of extrahepatic localization. The literature indicates that infection with *O*. *viverrini* can be accompanied by a clinical picture of dysbiosis, pain, and dyspeptic syndromes [9], implying an impact on organs outside the site of infection. Clinical studies have shown the progression of a renal pathology during infection with *O*. *viverrini* [10,11] and development of a renal pathology in an experimental model Golden hamsters (*Mesocricetus auratus*) [12].

Why does one species of closely related trematodes cause cancer, while another almost does not? The answer to this question may be determined not only by differences in the genome and biology but also by ecology and an accompanying microbiota. To answer this question, it is necessary to conduct comparative studies using experimental models in absence of additional environmental factors (such as differences in nutrition and microbiota) which may play a potentially important role. It should be noted that there are very few such studies now. Reason is long distance and difference between the habitats of these trematodes.

The aim of this study was to identify species-specific features of the development of liver and kidney pathologies in an experimental Syrian hamsters *Mesocricetus auratus* after infection with one of the three liver fluke species (*O*. *felineus*, *O*. *viverrini*, or *C*. *sinensis*) in an Animal Facility free from specific pathogens.

## 2. Material and methods

### 2.1. Ethics statement

All the procedures were in compliance with European Union Directive 2010/63/EU for animal experiments. Study design protocols and standard operating procedures (concerning the hamsters and the fish) were approved by the Committee on the Ethics of Animal Experiments at the Institute of Cytology and Genetics, the Siberian Branch of the Russian Academy of Sciences (ICG SB RAS) (Permit Number 126 of 3 August 2022).

### 2.2. Experiment animal and design

Forty male golden Syrian hamsters (*M*. *auratus*) from the Specific Pathogen-Free (SPF) Animal Facility at the ICG SB RAS were used for this study. All the procedures were performed aseptically at the SPF facility. The same investigator infected all the animals.

For collecting *O*. *felineus* metacercariae, naturally infected freshwater fish (*Leuciscus idus*) was net-caught in the Ob River near Novosibirsk, Western Siberia, Russia. *C*. *sinensis* and *O*. *viverrini* metacercariae were collected from naturally infected freshwater fish (Seoul, Republic of Korea, and Khon Kaen, Thailand, respectively) as described previously [13]. The metacercariae were transported in ice-cold PBS. The viability of metacercariae was assessed using microscope before infection.

**Fig 1:**
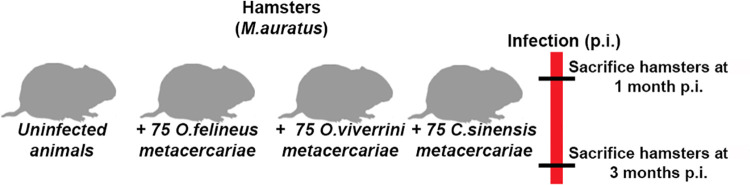
The experimental design. Infection of Syrian hamsters with 75 metacercariae of one of the three trematode species.

Forty hamsters were distributed into four groups, and animals from three of them were each infected with 75 metacercariae (of one of the three liver fluke species separately) by gastric intubation at interval of 3–5 days to avoid bacterial cross-infection. One group was kept uninfected as control. The experimental design is shown in Fig 1. Hamsters were kept under standard conditions and received rodent feed (PK-120-1; Laboratorsnab, LLC, Moscow, Russia) and water *ad libitum*. The hamsters were euthanized by isoflurane inhalation after 1 and 3 months postinfection (p.i.), and every effort was made to minimize their suffering.

### 2.3. Sampling of feces samples and examination

The stool samples from animals at 3 months p.i. were collected and stored at -80°C. Feces examination was performed using Mini PARASEP (Apacor Limited, UK) according to the manufacturer’s recommendations. Helminth eggs were identified using a microscope with 100X magnification, 100 microscopic fields were investigated per sample. For each sample, 3 technical replicates were carried out and the average value was calculated. Then the EPG (number of eggs per gram stool) was calculated by multiplying the number of eggs counted by the total volume of sediment in drops divide by the weight of feces in gram. It was found that hamsters infected with different trematode species had similar egg counts per gram (EPG). Thus, EPG for *O*. *felineus*-infected animals was 8523±3720; 7476±2559 for *O*. *viverrini*-infected animals, and 9957±3119 for *C*. *sinensis*-infected animals. No statistically significant differences were found between different trematode species.

### 2.4. Sampling of materials and histopathological analysis

Blood was collected by cardiac puncture, clotted and centrifuged at 3000 × *g* for 20 min at 4°C to obtain serum. The hamsters’ urine was collected by bladder puncture. The serum and urine samples were aliquoted and stored at −80°C. For histological analysis, the liver and kidney were resected and fixed in 10% buffered formalin (Biovitrum, Russia) for 7 days at 4°C. Three- to 4-μm-thick slices were prepared using a microtome (Microm, UK). The tissue slices were stained with hematoxylin–eosin (Biovitrum, Russia) and Masson’s trichrome dye (Biovitrum, Russia) by standard methods. The Periodic Schiff-Methenamine Silver (PASM) Kit (Biovitrum, Russia) was employed to stain the mesangial matrix on 2-μm paraffin sections of hamster kidneys according to the manufacturer’s protocol with following modifications. To improve the quality of glomerular-basement-membrane staining, iodic acid was replaced by 1% periodic acid. After silver impregnation, the sections were stained with eosin for 5 min. All slides were examined under an Axioplan 2 microscope (Zeiss, Germany). The following histological parameters were assessed: tubular casts in the lumen of the renal cortex and medulla, Bowman’s space, interstitial fibrosis, and the amount of the mesangial matrix per nephron glomerulus in kidneys as well as inflammatory infiltration, periductal fibrosis, epithelial hyperplasia, and biliary neoplasia (BiliN) in the liver.

Liver and kidney pathological changes were assessed by a scoring method of the ratio type [14] as described before [15,16]. In particular, all fields of view were analyzed (20–30 images from two liver lobes) in each animal. To assess structural change in kidney, 10 consecutive fields of view were selected. Each field of view was subdivided into 100 equal squares. The area of tubular casts was presented as a percentage of the number of squares occupied. The percentage of the area occupied by the mesangial matrix was calculated from the area of each glomerulus. For each hamster, 10 glomeruli were randomly chosen. Bowman’s space area was estimated according to the following algorithm: each capsule’s area was assigned a value from zero to 1.0, where zero is the absence of expansion, and 1.0 denotes capsule expansion by more than 15 μm. Each field of view was scored, and a mean score was assigned to the whole tissue of that animal [17]. Data were calculated using the ImageJ software (Bethesda, USA).

### 2.5. Immunohistochemistry

Immunohistochemical analysis was performed using a specific primary antibody: a polyclonal antibody to IgA *(1*:*150; cat*. *# PAA546Mu01Rb*, *Cloud Clone*, *China*), followed by staining with a secondary antibody: a goat anti-mouse IgG (H+L) antibody (*1*:*150; cat*. *# AS014*, *ABclonal*, *China*) according to the manufacturer’s protocol.

### 2.6. Serum and urine biochemical assays

Serum and urine alanine aminotransferase (ALT) and aspartate aminotransferase (AST) activity as well as cholesterol, creatinine, and triglyceride levels were measured with commercial kits (Vector-Best, Russia) in accordance with the manufacturer’s instructions. Serum KIM-1 (kidney injury molecule-1) concentration was assessed using an ELISA kit (Cloud-Clone Corp., China). Protein concentration in urine sample was quantitated using the BCA Protein Assay Kit (Thermo Fisher Scientific, USA).

### 2.7. Dot blot assay

For this assay, 10 μL of animal blood serum and urine were applied onto a nitrocellulose membrane (Bio-Rad, USA) at center of a square in grid and were dried at room temperature. The membrane was soaked in a 5% nonfat milk solution in TBS-T (20 mM Tris-HCl, 150 mM NaCl, and 0.05% of Tween 20) for 1 h at room temperature in order to block nonspecific binding. The membrane was washed once in TBS-T for 5 min and then incubated with the antibody to IgA *(1*:*500; cat*. *# PAA546Mu01Rb*, *Cloud Clone*, *China)* for 36 h at 4°C; next, the membrane was washed three times with TBS-T (3 × 5 min*)*. The membrane was incubated with a secondary antibody conjugated with horseradish peroxidase [the goat anti-mouse IgG (H+L) antibody (*1*:*7000; cat*. *# AS014*, *ABclonal*, *China*)] for 1 h at room temperature, followed by washing three times with TBS-T (3 × 5 min). The signal was detected with the ECL chemiluminescent reaction reagent (Amersham Biosciences, UK) on X-ray film. Quantitative densitometric analyses were performed on digitized images of dot blots in the Quantity One software (Bio-Rad, USA).

### 2.8. Statistical analyses

These analyses were performed in the STATISTICA 6.0 software (Statsoft, USA) and using the ggplot2‖ R package (version 4.1.0). Significant differences between the control and experimental groups of hamsters were evaluated by ANOVA + *post hoc* Tukey’s test. The time dependence of the pathological changes was assessed by one-way linear regression analysis. Significance of the differences between the groups of hamsters was evaluated by the Mann–Whitney test (quantitative histopathological analysis and serum biochemical parameters). Normality of a data distribution was determined by the Shapiro–Wilcoxon W test. Each graph was created using R packages ggplot2_3.5.0 and hrbrthemes_0.8.7 (version 4.1.0). P-value smaller than 0.05 (p<0.05) was considered statistically to be significant.

Five to 10 uninfected male hamsters aged 3 to 5 months were selected as an uninfected (control) group. After comparison of data from uninfected hamsters of different ages, we did not find significant age-related differences (ANOVA + *post hoc* Tukey’s test) either in biochemical parameters or in histological parameters (S1 Table). Thus, according to these findings, all uninfected animals were combined into one control group and used as a single control for all infected groups.

### 2.9. Bioinformatics analysis

Principal component analysis to evaluate clustering was performed using PCAtools_2.14.0 with the parameter scale = TRUE. Each graph was built using ggplot2_3.5.0 and hrbrthemes_0.8.7.

## 3. Results

### 3.1. Liver histopathology

The *C*. *sinensis* -infected animals’ livers were significantly enlarged at 1 and 3 months p.i. (P < 0.001 and <0.001, respectively), whereas with *O*. *felineus* infection, there was an enlargement only at 3 months p.i. (P < 0.05; S2 Table).

**Fig 2:**
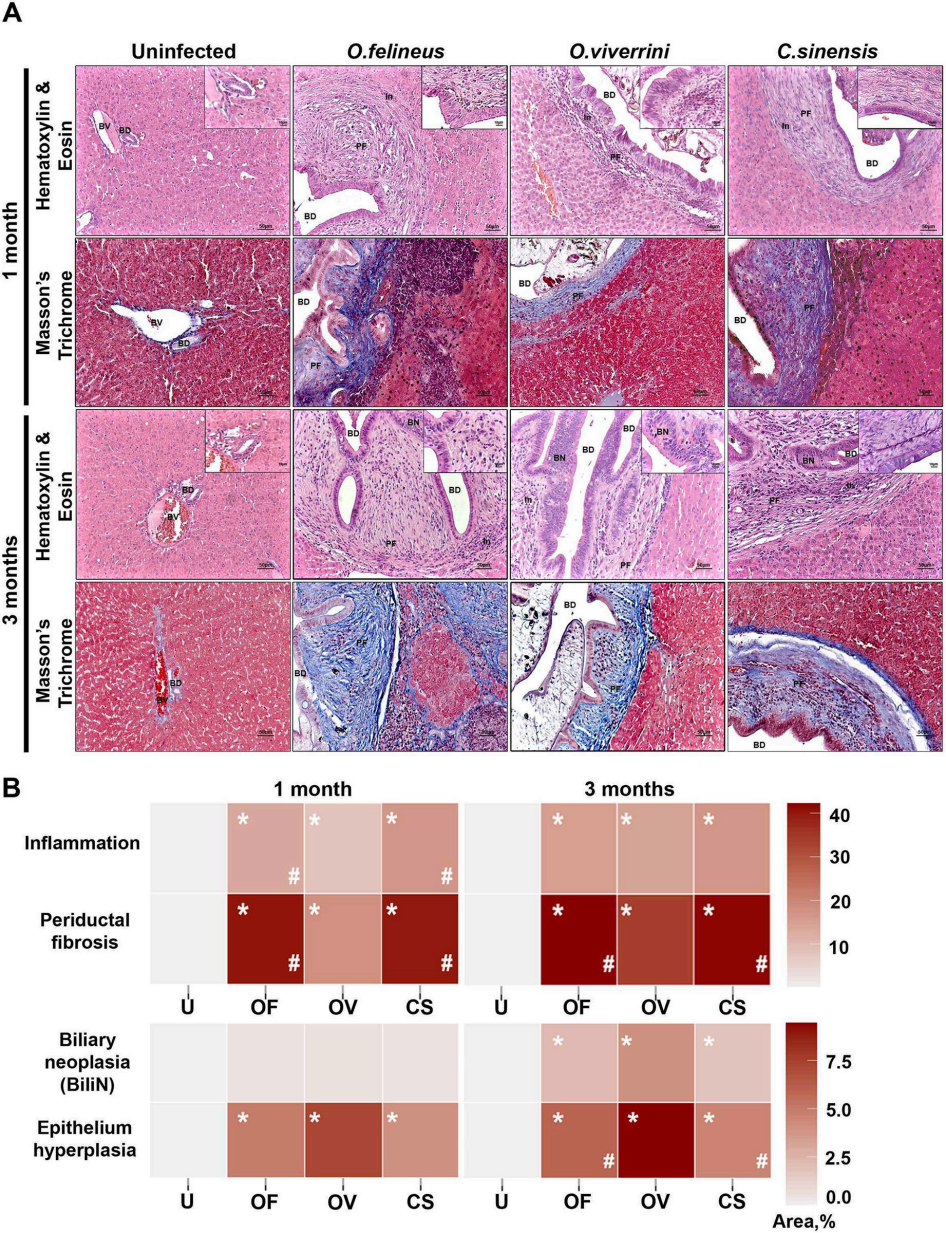
Pathologic findings in the *M*. *auratus* hamsters infected with three liver flukes. **A)** Liver sections stained with hematoxylin and eosin and Masson’s trichrome dye. **BD**: bile duct, **BV**: blood vessel, **PF**: periductal fibrosis, **BN**: biliary neoplasia, **In**: inflammation. **B)** Semiquantitative histological analysis of pathologic severity. The data are expressed as a percentage of a maximum possible score and are presented as a heatmap. *p < 0.05 as compared to the uninfected group, ^#^p < 0.05 as compared to the *O*. *viverrini* -infected group. U: uninfected animals; Of, Ov, and Cs: animals, infected with *O*. *felineus*, *O*. *viverrini*, or *C*. *sinensis*, respectively.

Analysis of liver sections of animals infected with one of the three liver fluke species: *O*. *felineus*, *O*. *viverrini*, or *C*. *sinensis* at 1 and 3 months p.i. showed pathological alterations specific for liver fluke infections. Both species-specific and time-dependent differences were identified (Fig 2A and 2B).

Inflammation levels in the *O*. *felineus* and *C*. *sinensis* -infected groups at 1 month p.i. were significantly higher compared with the uninfected group (P = 0.015 and 0.015) and the group infected with *O*. *viverrini* (P = 0.018 and 0.026, respectively). At 3 months p.i. (Fig 2; S3 Table), there were no significant differences between trematode species; however, inflammation was greater than that in the uninfected group.

Periductal fibrosis also had species-specific features, i.e., was different between groups. Already at 1 month p.i., all infected groups were different from the uninfected animals, consistently with literature data. At the same time, in hamsters infected with *O*. *felineus* or *C*. *sinensis*, within this period, greater periductal fibrosis was noted as compared to the *O*. *viverrini* -infected group (2-fold in the *O*. *felineus* -infected group, P = 0.012, and 2.6-fold in the *C*. *sinensis* -infected group, P = 0.006). At 3 months p.i., we observed a similar picture. Periductal-fibrosis area in the *O*. *felineus*—and *C*. *sinensis* -infected groups was 1.6 and 1.6 times larger than that in the *O*. *viverrini* -infected group (P = 0.011 and 0.011, respectively).

Bile duct proliferation was also most pronounced in *O*. *felineus*—and *C*. *sinensis* -infected groups at 1 month p.i. and was greater as compared with the group of *O*. *viverrini* -infected hamsters (P = 0.001 and 0.002, respectively). Although at 3 months p.i., bile duct proliferation during infection with *O*. *viverrini* increased relative to uninfected animals (P = 0.021), it remained less than this parameter observed during infection with *O*. *felineus* by 1.9-fold (P = 0.004) and *C*. *sinensis* by 1.7-fold (P = 0.005).

In contrast, in the bile duct epithelium, changes were most pronounced in the *O*. *viverrini* -infected group. For instance, the level of epithelial hyperplasia in the *O*. *viverrini* group at 1 month p.i. exceeded this level in other infected animals by 1.9-fold (P = 0.018) and 2.4-fold (P = 0.05) as compared with the *O*. *felineus* -infected and *C*. *sinensis* -infected groups, respectively. A similar pattern was observed at 3 months p.i.: the epithelial-hyperplasia level during *O*. *viverrini* infection was 1.9 times higher (P = 0.026) than that in the *O*. *felineus* -infected group, and 2.45 times (P = 0.002) higher as compared with the *C*. *sinensis* -infected group. The BiliN magnitude was significantly different from the control only at 3 months p.i. in all infected groups, and no significant differences were detectable between different trematode species (Fig 2B and S3 Table).

**Fig 3:**
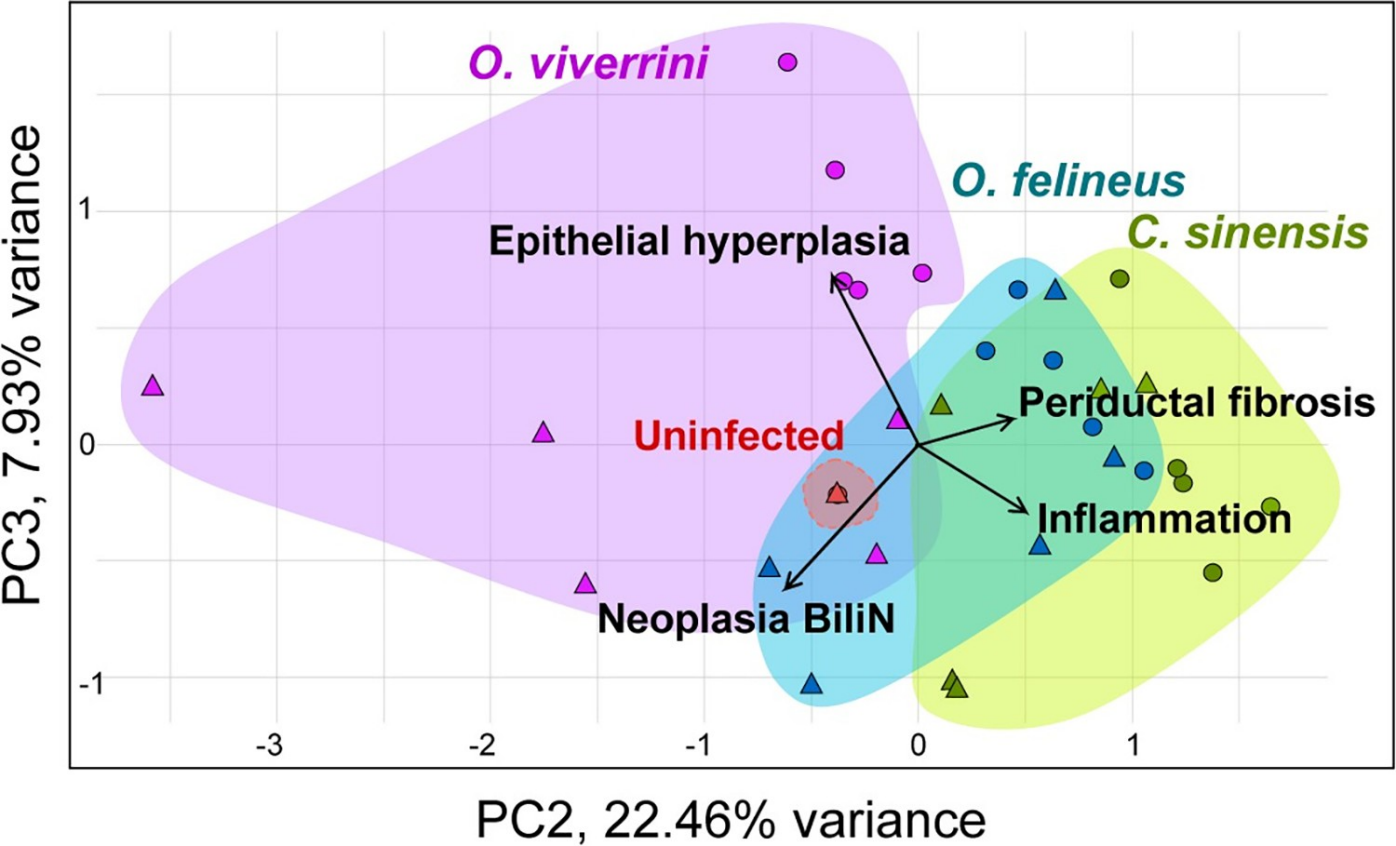
Clustering of liver samples according to principal component analysis based on semiquantitative histopathology data. **PC-** principal component**, BiliN**–biliary intraepithelial neoplasia.

From the obtained data, it can be inferred that the patterns of development of the infection caused by *O*. *felineus* and *C*. *sinensis* are similar and are more profibrotic, in comparison to *O*. *viverrini* infection. Principal component analysis based on semiquantitative histological analysis showed clustering of *O*. *viverrini* -infected animal samples separately from the other groups. The reason is probably the development of pathological changes in the epithelium of bile ducts. It is worth noting that *O*. *viverrini* samples at 1 and 3 months p.i. clustered separately from each other. This effect is likely related to the development of BiliN. Samples from groups of animals infected with *O*. *felineus* and *C*. *sinensis* clustered together due to similar dynamics of periductal fibrosis (Fig 3).

### 3.2. Liver and kidney biochemical parameters

Several species-specific changes in biochemical parameters of blood serum and urine were identified in infected animals. The triglyceride concentration in the blood serum of animals infected with *O*. *viverrini* after 3 months was found to be elevated by 1.9-fold (P = 0.007) and—in contrast to the other infected groups—was significantly higher than this concentration in uninfected animals. Although there was an increase in the level of proinflammatory cytokines IL-6, IL-17, and TNFα in all infected groups, significant changes were noted only in the *O*. *viverrini* -infected group at 1 month p.i. (IL-6 level increased 3.8-fold, P = 0.05, TNFα level increased 7.4-fold, P = 0.05) and in the *O*. *felineus* -infected group at 3 months p.i. (TNFα level increased by 7.4-fold, P = 0.05).

An important result was changes in biochemical markers of the functional state of kidneys. For instance, in the groups infected with *O*. *felineus* or *O*. *viverrini*, the creatinine level in the blood serum of hamsters proved to be elevated at 1 month p.i. by 2-fold (P = 0.029) and 1.9-fold (P = 0.017), respectively. Nonetheless, there were no differences in the serum creatinine level between the *C*. *sinensis* -infected group and uninfected group.

After 3 months of infection, the serum creatinine concentration was found to be increased in all infected groups: 1.8–2.0-fold. Creatinine in urine was elevated after 1 month in the *O*. *felineus*—and *C*. *sinensis* -infected groups by 5.2-fold (P = 0.014) and 2.5-fold (P = 0.008), respectively.

**Fig 4:**
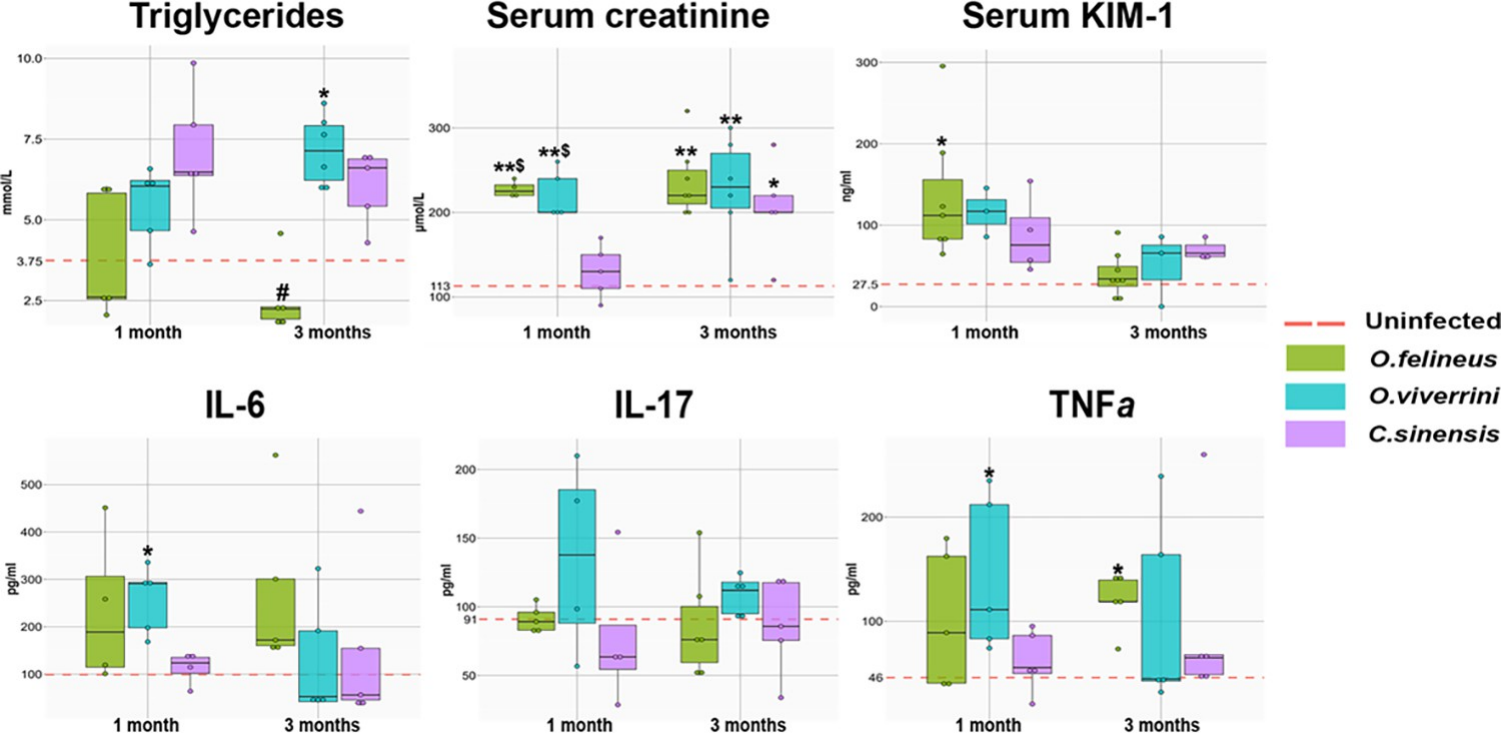
Serum biochemical parameters of hamsters infected with *O*. *felineus*, *O*. *viverrini* or *C*. *sinensis* at 1 and 3 months p.i. KIM-1: kidney injury molecule 1. **TNFa**: tumor necrosis factor alpha. IL: Interleukin. *p < 0.05 as compared to the uninfected group, ^$^p < 0.05 as compared to the *C*. *sinensis* -infected group.

The KIM-1 protein concentration in the blood serum of uninfected animals was 45.1 ± 24 mg/L. At 1 month p.i., the concentration of KIM-1 in the blood serum of *O*. *felineus* -infected animals was significantly (P = 0.02) higher than the control level by 3-fold (Fig 4 and S4 Table).

The above changes in biochemical parameters showed the development of a renal pathology, which among other things, could be species-specific. To assess the severity of kidney damage, a comparative histological analysis of this organ’s sections was carried out.

### 3.3. Kidney histopathological examination

Based on a previous histopathological analysis of *O*. *felineus* -infected hamsters’ kidneys [12], the following parameters were selected: expansion of Bowman’s space, tubular casts in the medulla and cortical layers of the kidneys, and interstitial fibrosis.

An expansion of Bowman’s space in *C*. *sinensis* -infected animals was noted at 1 month p.i. and was 1.8 times greater than that in the uninfected group (P = 0.019). At 3 months p.i., significant differences were observed in each infected group. Bowman’s space enlarged by 2.1-fold (P = 0.006) in the *O*. *felineus* -infected group, by 2-fold in the *O*. *viverrini* -infected group (P = 0.006), and by 1.7-fold in the *C*. *sinensis* -infected group (P = 0.009). Nevertheless, no significant differences were found among the infected groups.

**Fig 5:**
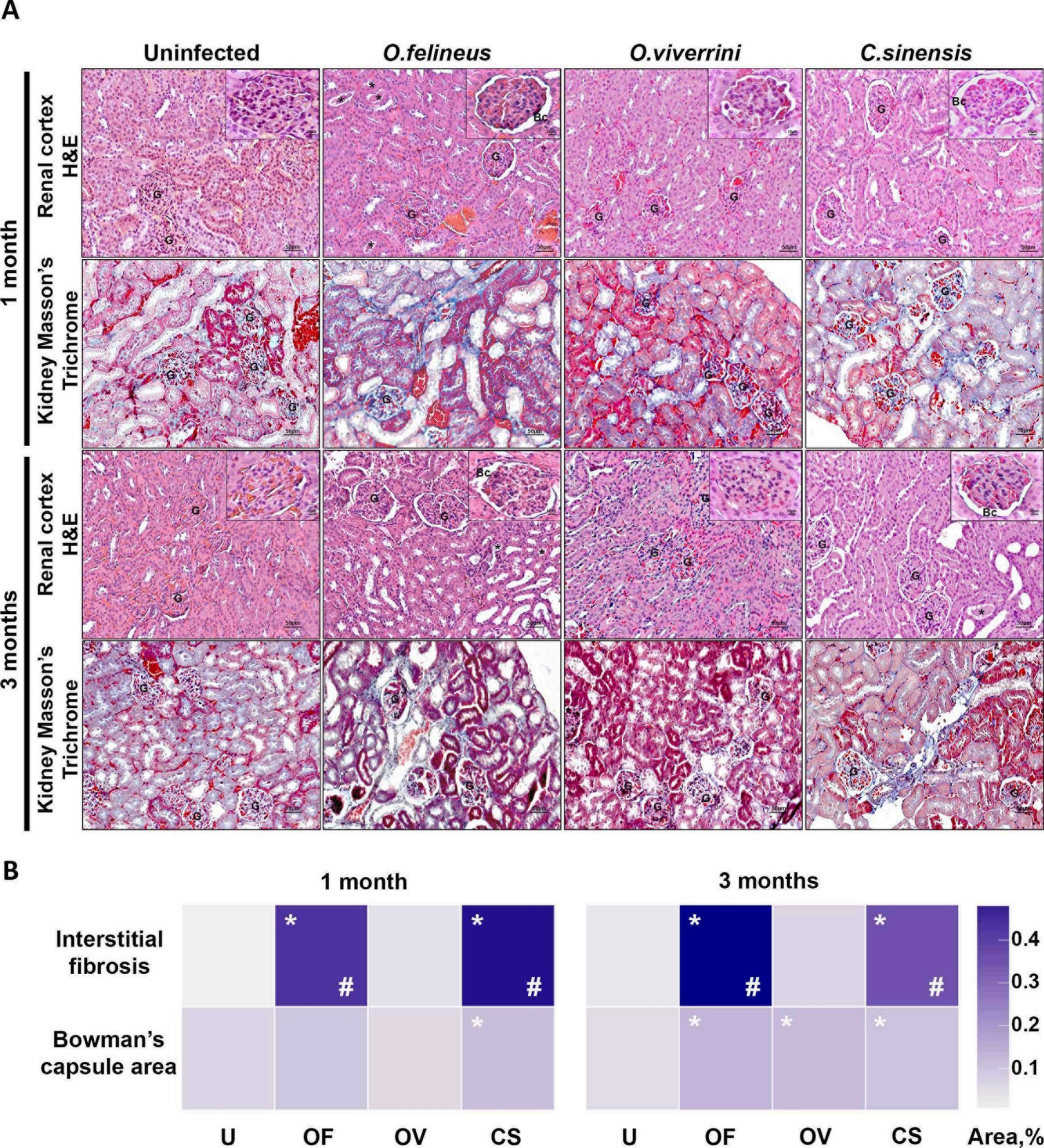
Kidney histopathology in *M*. *auratus* hamsters uninfected or infected with O. felineus, O. viverrini or C. sinensis at 1 and 3 months p.i. **A)** Kidney sections from *M*. *auratus* hamsters at 1 and 3 months p.i. Kidney sections were stained with hematoxylin and eosin and Masson’s trichrome dye. **B)** Quantification of morphological kidney alterations after liver fluke infection. The data are expressed as a percentage of a maximum possible score and are presented as a heatmap. *p < 0.05 as compared to the uninfected group, ^#^p < 0.05 as compared to the *O*. *viverrini* -infected group. U: uninfected animals; Of, Ov, and Cs: animals infected with *O*. *felineus*, *O*. *viverrini*, or *C*. *sinensis*, respectively. **G:** glomerulus; *casts; **IF**: interstitial fibrosis, **Bc**: Bowman’s capsule space.

An increase in the KIM-1 protein level points to tubular-epithelium damage. Furthermore, small loci of interstitial fibrosis were detected in hamsters’ kidney tissues after infection with any trematode species. The most pronounced signs of interstitial fibrosis were registered in the *O*. *felineus* -infected group at 1 and 3 months p.i. and exceeded this parameter of the control group by 21.5-fold (P = 0.0206) and 25.5-fold (P = 0.0079), respectively (Fig 5 and S5 Table).

In the *C*. *sinensis* -infected group, a 26.5-fold (P = 0.0084) increase in the area of interstitial fibrosis was noted at 1 month p.i. as compared with the uninfected group. At the same time, this parameter in the *O*. *felineus* -infected and *C*. *sinensis* -infected groups at 1 month p.i. exceeded that in the *O*. *viverrini* -infected group by 10.7-fold (P = 0.05) and 12.7-fold (P = 0.05), respectively. There were no significant changes in the area of interstitial fibrosis in *O*. *viverrini* -infected hamsters (Fig 5 and S5 Table).

Moreover, histological aberrations of the epithelium of proximal and distal tubules were detected in the kidneys. Epithelial cells were smaller, and the density of cells relative to each other was higher. Their nuclei were more condensed but retained a rounded shape. The lumen of these tubules was found to be significantly narrowed or completely absent. There were signs of glomerulofibrosis at 3 months p.i. in *C*. *sinensis*—and *O*. *felineus* -infected groups but not in the *O*. *viverrini* -infected group (Fig 5 and S5 Table).

### 3.4. The mesangial matrix and IgA levels

The amounts of the mesangial matrix in the kidneys at 1 and 3 months p.i. were different between the three trematode infections. The expansion of the mesangial matrix in glomeruli at 1 month p.i. was weak in all groups, and at 3 months p.i., it exceeded the level of the control group (20.4 ± 3.21) by 1.2-fold in the *O*. *felineus* -infected group (P = 0.0303) and by 1.5-fold in the *O*. *viverrini* -infected group (P = 0.0043). In the *C*. *sinensis* -infected group, expansion of the mesangial matrix was observed too; however, probably due to substantial individual differences between hamsters, the changes were not significant. One of the common reasons for mesangial area expansion may be the deposition of immunoglobulins, in particular IgA. Therefore, we performed immunohistochemical analysis of IgA in kidney sections.

**Fig 6:**
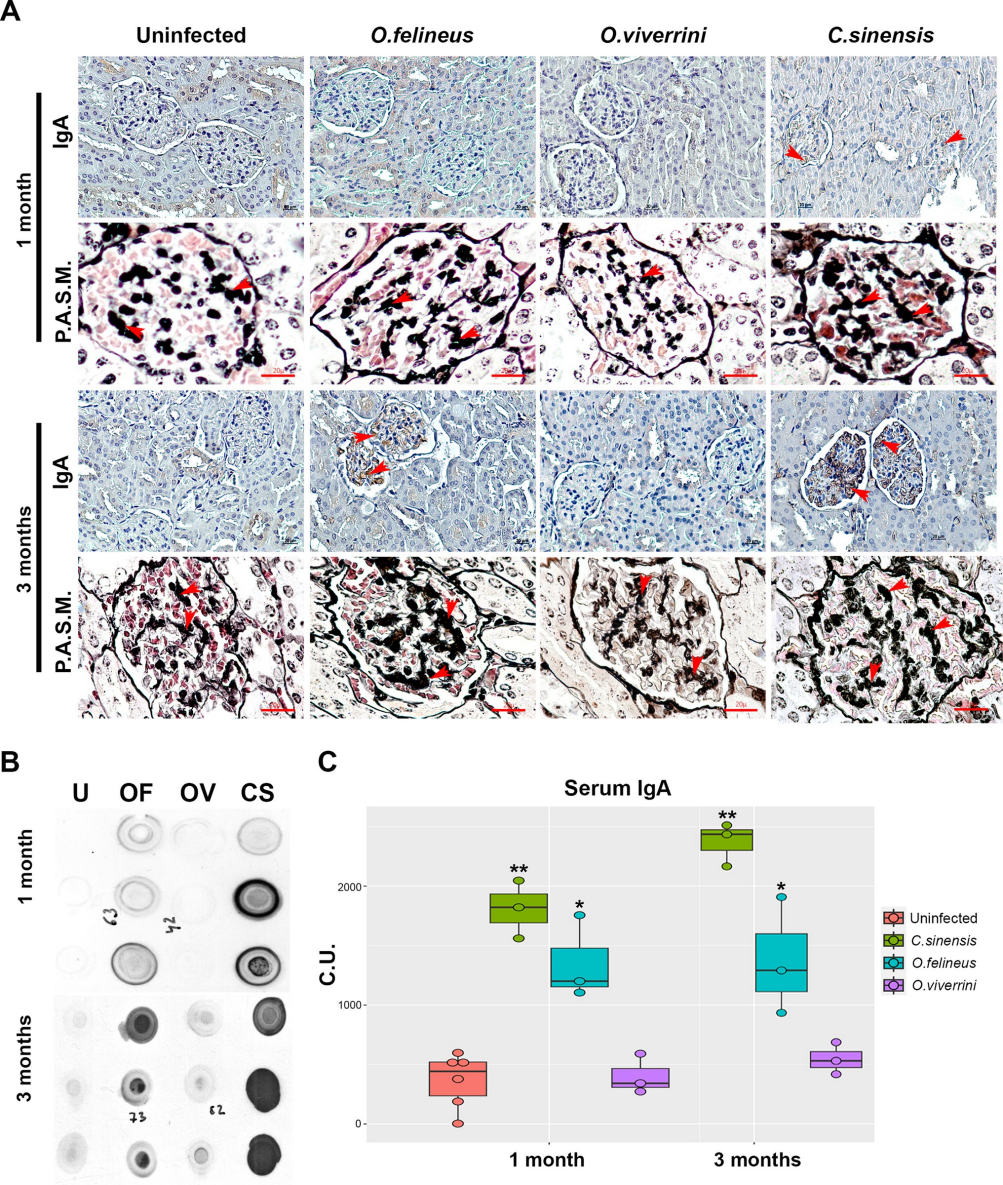
IgA nephropathy in liver fluke–infected hamsters at 1 and 3 months p.i. **A)** Kidney pathology according to IgA and silver staining. **B)** Representative dot blot images of IgA in the serum of hamsters. **C)** Quantification of dot blots. OF: *O*. *felineus*; OV: *O*. *viverrini*; CS: *C*. *sinensis*; P.A.S.M.: Periodic Schiff-Methenamine Silver; C.U.: conventional units.

IgA deposits were registered in glomeruli of *O*. *felineus* -infected animals at 3 months p.i. and in *C*. *sinensis* -infected animals at 1 and 3 months p.i. IgA deposits in kidney glomeruli of *O*. *viverrini* -infected hamsters were minimal. A specific signal was detected directly in the glomeruli, in the region of mesangial cells, but in a few cases, it was found in epithelial cells of renal tubules. In addition, the IgA content of the blood serum in hamsters proved to be increased in *C*. *sinensis* -infected animals at 1 month (P = 0.018) and 3 months (P = 0.005) p.i. by 3.5- and 6-fold, respectively, and in the group of *O*. *felineus* -infected animals at 1 and 3 months p.i. (P = 0.05) by 2.6-fold and 3.5-fold, respectively. At the same time, in uninfected hamsters, there were no signs of IgA accumulation in either kidney tissue or blood serum. The data are presented in Fig 6.

**Fig 7:**
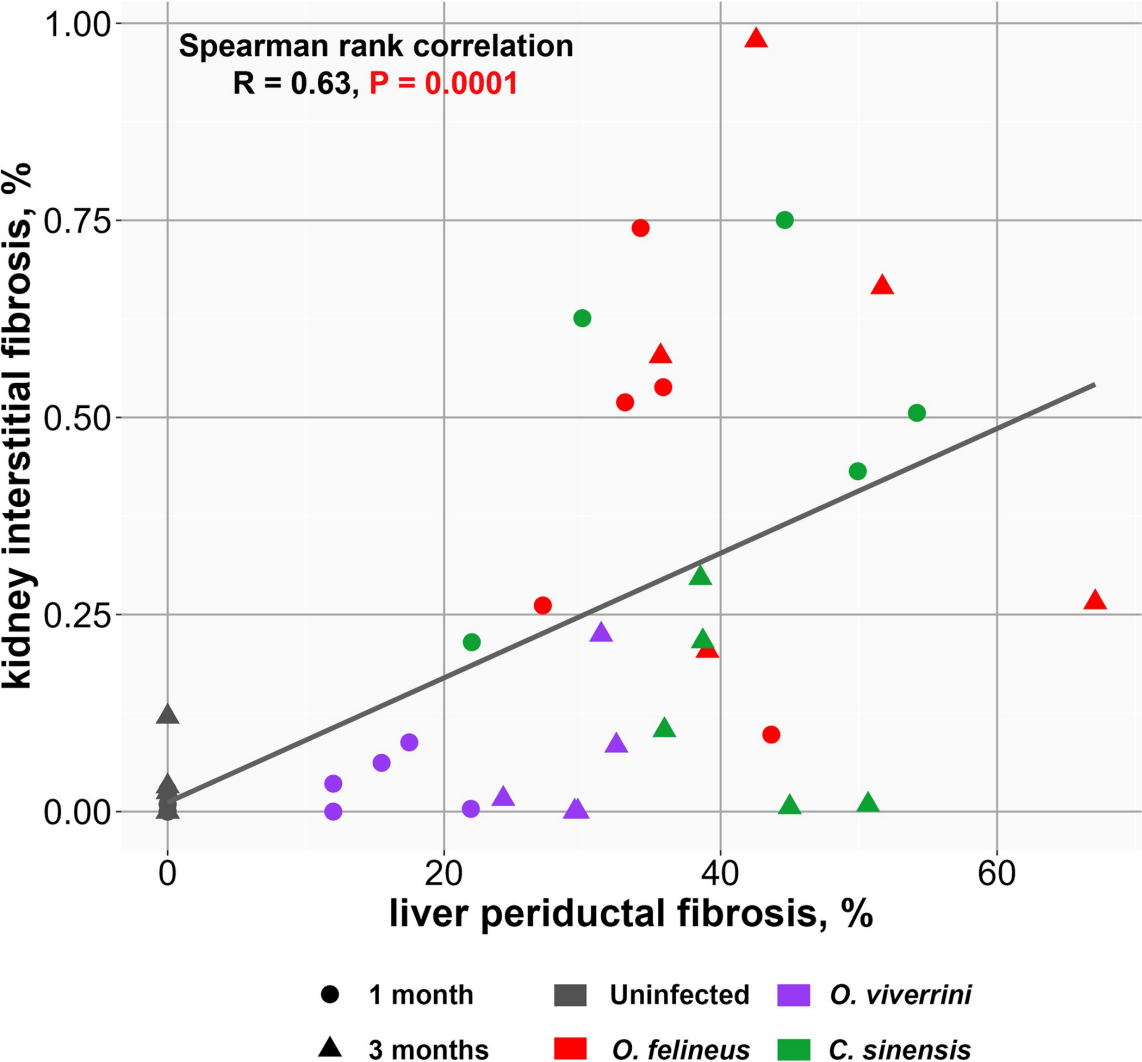
Spearman rank correlation analysis of the degree of fibrotic aberrations in the kidneys and liver. A relation between the severity of kidney interstitial fibrosis and liver periductal fibrosis. **OF:** *O*. *felineus***; CS:** *C*. *sinensis***; OV:** *O*. *viverrini*.

To evaluate the link between liver damage and kidney damage, we performed a correlation analysis. A significant correlation was detected between levels of liver periductal fibrosis and kidney interstitial fibrosis (R = 0.63, S = 4272, P = 0.0001) (Fig 7). Thus, a direct link was identified between the levels of liver fibrosis and kidney fibrosis.

## Discussion

In this study, for the first time, we show different directions of early pathological changes in three liver fluke infections. On the one hand, fibrogenesis and inflammation in the liver were more pronounced in *C*. *sinensis* -infected and *O*. *felineus* -infected groups; on the other hand, bile duct epithelium hyperplasia and BiliN were greater in *O*. *viverrini* -infected hamsters. The elevated level of hyperplastic and neoplastic changes in the bile duct epithelium in *O*. *viverrini* infection is in good agreement with indirect data on its highest carcinogenic potential among all liver fluke infections, given that BiliN is considered a precarcinogenic epithelium alteration.

One of possible consequences of liver fluke infection is a malignant tumor of the biliary epithelium and cholangiocarcinoma: one of the most malignant human tumors. Liver flukes *O*. *viverrini* and *C*. *sinensis* are recognized by the International Agency for Research on Cancer [8] as Group 1 biological carcinogens and are classified as major risk factors of cholangiocarcinoma in endemic areas, while *O*. *felineus* is classified as a Group 3 agent: a noncarcinogen for humans [8]. It is unknown what exactly causes the differences in the carcinogenic potential between *O*. *felineus*, *O*. *viverrini*, and *C*. *sinensis* [2].

It is noteworthy that some differences between the pathologies caused by these three species have been demonstrated previously. For example, in a comparison of the pathogenesis between *O*. *viverrini* and *O*. *felineus* infections in conventional animals, more inflammatory infiltrates were noted in *O*. *felineus* infection than in *O*. *viverrini* infection [18], consistently with our data. Differences in pathogenesis among liver fluke infections have also been observed in the microbiome of animals infected with different species of trematodes [13]. For instance, *O*. *viverrini* infection modifies bile microbiome communities extensively, whereas *O*. *felineus* infection results in the smallest changes. Differences among the three infections may also be due to biological characteristics; in particular, the expression of several dozen genes is species-specific in transcriptomes of adult worms of *O*. *felineus*, *O*. *viverrini*, and *C*. *sinensis* [19].

Probably, the differences in the effects of the trematodes on the host may also be due to the activation of dissimilar signaling pathways during the host response. For example, we showed that proinflammatory cytokines IL-6 and TNFα are upregulated in *O*. *felineus* -infected and *O*. *viverrini* -infected hamsters to a greater extent than in *C*. *sinensis* -infected animals, while no significant changes in the level of IL-17 were detectable. Proinflammatory cytokines IL-1, IL-6, TNFα, and IL-17 play an important role in the regulation of inflammation and are capable of stimulating proliferative and antiapoptotic signals in epithelial and tumor cells or inducing angiogenesis [20]. Upregulation of these cytokines has been documented during parasitic infections [21,22]; however, in some cases, a time dependence has been observed in these changes, and our results do not completely support those obtained previously [21–23]. We can hypothesize that the discrepancies may be explained by the timing of infection [23] or by the contribution of other variables, including environmental factors and differences between SPF and conventional animals.

In our study, inflammation and periductal fibrosis in the liver were accompanied by deteriorating liver function, judging by activity of major liver transaminases and by bilirubin levels, consistently with previous data [21,24,25].

An important finding was changes in biochemical markers of the kidney functional state, including overexpression of creatinine and KIM-1 in the blood serum of infected animals, consistently with previously obtained literature data [12,26] and our current results. Changes in biochemical parameters of renal function here were accompanied by changes in renal morphology, in particular, by kidney interstitial fibrosis and expansion of Bowman’s space. Bowman’s capsule is a structural part of the nephron and forms a cup-shaped sac around the glomerular tuft, and its expansion may be an early warning sign of severer pathological changes [27]. On the other hand, kidney fibrosis was more pronounced during infection with *O*. *felineus* and *C*. *sinensis*. Nevertheless, minor interstitial fibrosis loci were found in *O*. *viverrini -*infected hamsters too.

Because the levels of fibrosis in both kidneys and liver were greater during infection with *O*. *felineus* or *C*. *sinensis*, we hypothesized a close relation between these alterations. To detect such a relation, we performed a correlation analysis between liver periductal fibrosis and kidney interstitial fibrosis. The results confirmed our hypothesis: the larger area of periductal fibrosis in the liver, the more pronounced is interstitial fibrosis in the kidneys.

The literature contains evidence of the development of kidney disease in humans during various infections, including dengue hemorrhagic fever, typhoid fever, shigellosis, leptospirosis, malaria, schistosomiasis [28–30], and opisthorchiasis caused by *O*. *viverrini* [31] or *O*. *felineus* [32,33]. It should be pointed out that such a relation has previously been demonstrated in schistosomiasis. In particular, it has been shown that membranoproliferative and focal segmental glomerular sclerosis correlate with liver fibrosis, while IgA plays an important part in their pathogenesis [34]. Therefore, we turned our attention to the expansion of the mesangial matrix and IgA accumulation in the kidneys and blood serum of hamsters infected with *O*. *felineus* or *C*. *sinensis*.

In this work, we noticed expansion of the mesangial matrix at 3 months p.i. in all infected groups. In most cases, expansion of the mesangial matrix and hence the development of mesangioproliferative glomerulonephritis are strongly associated with the deposition of IgA in glomeruli [35]. These abnormalities are also known as IgA nephropathy. The latter is characterized by focal or diffuse proliferation of the mesangial matrix and can lead to severe kidney injury, such as interstitial fibrosis, capillary necrosis in glomeruli, and renal tubular atrophy [36,37].

According to published data, there have been cases of accumulation of circulating immune complexes in the glomerular filter during parasitic infections [38]. For example, a kidney pathology was documented during infection with *S*. *haematobium* or *S*. *mansoni*, where renal damage was noted as interstitial nephritis, tubular dysfunction syndrome, and deposition of the immune complex of a schistosome antigen with IgG/IgM antibodies in the glomerular basement membrane, which led to different types of glomerulonephritis [39,40]. Mesangioproliferative glomerulonephritis and membranoproliferative glomerulonephritis have also been reported that are caused, among other things, by circulating immune complexes in animals infected with *Fasciola hepatica* [41,42]. Similarly, after *O*. *viverrini* (OV) infection, the development of a renal pathology has been demonstrated that is accompanied by mesangial proliferative glomerulonephritis with immune complexes consisting of IgG, complement component 3 (C3), and an *O*. *viverrini* antigen [43]. As shown later, however, disturbances in the structural and functional state of kidneys during *O*. *viverrini* infection are typical for later stages of infection [25,10], consistently with our data.

Although a kidney pathology has been detected in various parasitic infections and in diseases of a noninfectious nature [44], in particular nonalcoholic fatty liver disease [45], the mechanisms are still unknown. The reason is probably the lack of a direct relation between hepatobiliary and renal pathologies. A renal pathology, in particular tubulopathy, can develop as a consequence of cholestasis of various etiologies as well as in response to acute liver damage [46].

Thus, we present convincing evidence of a strong correlation between liver fluke infection and kidney damage, judging by the development of interstitial fibrosis in the kidneys, expansion of the mesangial matrix, and IgA deposition.

A comparative analysis of human diseases caused by the closely related species of food-borne trematodes *O*. *felineus*, *O*. *viverrini*, and *C*. *sinensis* (which have different geographical ranges) seems difficult. The comparative research is also complicated by a number of factors, including climatic features, the history of a nation, its economy, its cultures, traditions, nutritional habits, alcohol consumption and smoking, and various concomitant infections, including *H*. *pylori* and others. In addition to the above factors that can influence the pathogenesis of infection, the worm load level and susceptibility of laboratory animals are important. Thus, according to available published data, rats are considered to be a susceptible laboratory model for *C*. *sinensis*, but not for *O*. *viverrini* [47,48]. However, according to our and published data, hamsters are a susceptible laboratory model for all three species of liver flukes [49]. To assess the level of infection, we estimated egg number in hamsters’ feces. A similar number of eggs in feces serves as an indicator of both the sexual maturity of helminths and an indicator of a similar worm load, which is important for the consequences on pathogenesis [50,51]. At the same time, the size of adult worms differs. It is known that the biggest physical size of adult worm has *C*.*sinensis*, the smallest—*O*. *viverrini*. However, to our knowledge there are no data on the relationship between the size of each of the three trematodes species and the severity of pathological changes. Additionally, results obtained on laboratory animals are difficult to compare if they have been obtained in different experimental studies that do not take into account housing and management of laboratory animals in conventional animal facilities, human factors, concomitant infections of laboratory animals, and other confounding variables. Consequently, the amount of available data remains sparse in this field and makes it difficult to draw conclusions about differences in pathogenicity among the three closely related species of liver flukes. Therefore, an advantage of our work is investigation of differences in pathologies among hamsters infected with *O*. *felineus*, *O*. *viverrini*, or *C*. *sinensis*, under identical conditions, at the same time, and at an SPF animal facility (free from specific pathogens). This approach allowed us to minimize numerous confounding factors.

According to the current understanding of fibrogenesis, liver inflammation can precede the development of events via one of the following scenarios: tumorigenesis or fibrosis [52]. The comparison of three infections having different carcinogenic potentials serves as an adequate model for studying key processes behind shifts of the metabolic balance in one of the two directions and will help answer the question of what underlies biological carcinogenesis. In this study, we demonstrated pronounced differences in the pathophysiology between infections caused by three opisthorchid species in an experimental model. Nonetheless, we cannot yet name the mechanisms underlying the different directions of the disease course: toward profibrotic or toward oncogenic alterations in the liver. For this purpose, apparently, it is necessary to investigate the cellular pathways and regulatory signaling cascades that are activated during liver fluke infection, cause metabolic reprogramming of the liver, and contribute to biological carcinogenesis, i.e., carcinogenesis associated with liver flukes.

It is worth noting that in our study, the species specific response was assessed at 1 and 3 months postinfection. These time points reflect the changes that occur during the transition between acute and chronic infection, including early events in the development of BILIN. This study did not investigate longer term study outcomes. The development of serious complications during the long term of the disease, as well as under the influence of additional factors, including concomitant infections and the use of dimethylnitrosamine [9,53–57] should be investigated in future studies to understand the mechanisms underlying the liver fluke-associated carcinogenesis.

## Supporting information

S1 Fig Endemic area of *Opisthorchis felineus*, *O*. *viverrini* and *Clonorchis sinensis*. (TIF)

S1 Table Histopathological and biochemical analysis of uninfected hamsters’ parameters. (DOCX)

S2 Table Liver and kidney weights of hamsters infected with one of the three liver fluke species. (DOCX)

S3 Table Liver histopathological analysis. The values used to build heatmap at Fig 2B.
(DOCX)

S4 Table Serum biochemistry. (DOCX)

S5 Table Kidney histopathological analysis. The values used to build heatmap at Fig 5B.
(DOCX)
