# Supplementary figures and images for: Species-specific renal and liver responses during infection with food-borne trematodes Opisthorchis felineus, Opisthorchis viverrini, or Clonorchis sinensis
Source: PLoS One. 2024 Dec 5;19(12):e0311481. doi: 10.1371/journal.pone.0311481 (PMC11620611; doi:10.1371/journal.pone.0311481)

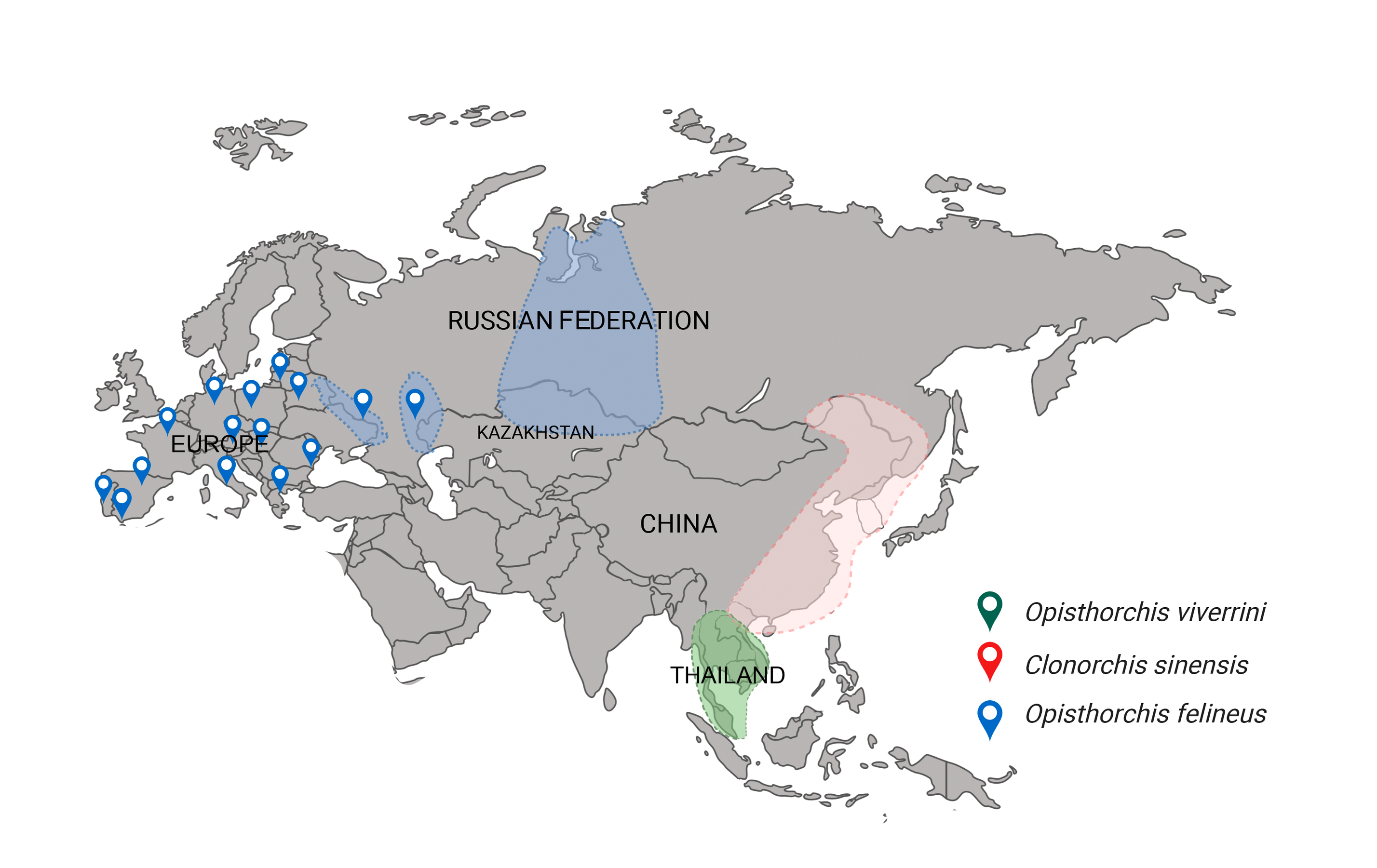

Supplement: S1 Fig — (TIF) [file pone.0311481.s001.tif]
