# Supplementary material for: Species-specific renal and liver responses during infection with food-borne trematodes Opisthorchis felineus, Opisthorchis viverrini, or Clonorchis sinensis
Source: PLoS One. 2024 Dec 5;19(12):e0311481. doi: 10.1371/journal.pone.0311481 (PMC11620611; doi:10.1371/journal.pone.0311481)
Supplement: S1 Table — (DOCX) [file pone.0311481.s002.docx]

**Supplementary Table 1. Histopathological and biochemical analysis of uninfected hamster liver, kidney, serum and urea at the age of 3 and 5 months (Mean±SD).**

| **Histological changes in the liver** |  | |
| --- | --- | --- |
|  | **1 month** | **3 months** |
| Inflammation | 0.06±0.13 | 0.05± |
| Periductal fibrosis | 0 | 0 |
| Bile duct proliferation | 0 | 0 |
| Epithelium hyperplasia | 0 | 0 |
| Biliary neoplasia | 0 | 0 |
| **Histological changes in the kidney** |  |  |
|  | **1 month** | **3 months** |
| Bowman’s space area | 0.05 ± 0.02 | 0.06 ± 0.02 |
| Tubular casts in cortex | 0.01 ± 0.01 | 0.01±0.01 |
| Tubular casts in medulla | 0.02 ± 0.03 | 0.02 ± 0.03 |
| Mesangial matrix | 22.5 ± 4 | 20.1 ± 3.2 |
| Interstitial fibrosis | 0.009 ± 0.01 | 0.03 ± 0.04 |
| **Biochemical changes** |  |  |
|  | **1 month** | **3 months** |
| ALT (U/L) | 21.1 ± 10.4 | 35 ± 8.5 |
| AST (U/L) | 24 ± 17.4 | 40.6 ± 31.3 |
| Total cholesterol, (mmol/L) | 2.2 ± 0.9 | 3.2 ± 0.6 |
| Triglycerides, (mmol/L) | 2.8 ± 1.3 | 4.4 ± 2.6 |
| Urine protein (mg/L) | 0.7 ± 0.2 | 1.23 ± 0.4 |
| Urine creatinine (µmol/L) | 89.1 ± 17.2 | 58.8 ± 50.63 |
| Serum KIM-1 (mg/L) | 27.5 ± 3 | 45.1 ± 24 |

P values were obtained by the Mann–Whitney U test.

For histopathological analysis, the tissue slides were stained with hematoxylin and eosin or Masson’s Trichrome. Histological features of liver were manually scored by two independent investigators and a senior pathologist confirmed the score. A scoring method of type Ratio (morphometry) of data measurements was applied (Gibson-Corley, 2013). This method is based on counting several fields of tissue (e.g. ten random 400x fields) for each animal, each field scored and a mean score assigned for the whole tissue of that animal. Each slice of a lobe was analyzed in all fields of view (20–30 fields). Each field of view was divided into 100 squares. Inflammatory cell infiltration, cholangiocyte hyperplasia, Biliary neoplasia, periductal fibrosis, and bile duct cell proliferation were assessed by means of a percentage of the area (the number of squares occupied).

To assess structural kidney changes, 20 random fields of view (x200 magnification) were selected. Each field of view was subdivided into 100 equal square units (conditional units) in the ImageJ software. The area of the casts is presented as a percentage of the number of squares occupied. The percentage of the area occupied by the mesangial matrix was calculated from the area of each glomerulus. For each animal, 10 glomeruli were randomly chosen. Bowman’s space area was estimated according to the following algorithm: each capsule space area was assigned a value from zero to 1.0, where zero is the absence of expansion, and 1.0 denotes capsule expansion by more than 15 μm. Each field of view was scored, and a mean score was assigned to the whole tissue of that animal (Klopfleisch, 2013).
