# Supplementary material for: Species-specific renal and liver responses during infection with food-borne trematodes Opisthorchis felineus, Opisthorchis viverrini, or Clonorchis sinensis
Source: PLoS One. 2024 Dec 5;19(12):e0311481. doi: 10.1371/journal.pone.0311481 (PMC11620611; doi:10.1371/journal.pone.0311481)
Supplement: S2 Table — (DOCX) [file pone.0311481.s003.docx]

**Supplementary Table 2. Liver and kidney weights and relative liver and kidney weights of *M. auratus* hamsters infected with one of the three liver fluke species.**

| **Group** | **No. of hamsters** | **Body weight**  **(mean±SD)** | **Liver**  **Weight**  **(mean±SD)** | **Relative liver weight**  **(mean±SD)** | **Kidney weight**  **(mean±SD)** | **Relative kidney weight**  **(mean±SD)** |
| --- | --- | --- | --- | --- | --- | --- |
| **Uninfected** | 18 | 115.37 ± 11.39 | 4.05 ± 0.78 | 0.034 ± 0.005 | 1.106 ±0.22 | 0.009±0.002 |
| ***O. felineus* 1 month** | 5 | 116.32 ± 4.052 | 4.602 ± 0.215^$^ | 0.039 ±0.001*^$$$^ | 1.1 ± 0.082 | 0.009 ± 0.0006 |
| ***O. viverrini* 1 month** | 5 | 115.32 ± 11.47 | 3.886 ± 0.374^$$$^ | 0.033 ± 0.0009^$$$^ | 1.05 ± 0.092 | 0.009 ± 0.0004 |
| ***C. sinensis* 1 month** | 5 | 114.51 ± 10.347 | 6.09 ± 0.495*** | 0.053 ± 0.005***^@@@###^ | 0.92 ± 0.203 | 0.007 ± 0.0013 |
| ***O. felineus* 3 months** | 5 | 119.36 ± 9.228 | 5.333 ± 0.692**^$$$^ | 0.044 ± 0.002***^$$$^ | 1.042 ± 112 | 0.008 ± 0.0004 |
| ***O. viverrini* 3 months** | 5 | 117.35 ± 10.377 | 4.491 ± 1.029^$$$^ | 0.038 ± 0.006^$$$^ | 0.993 ± 0.104 | 0.008 ± 0.0006 |
| ***C. sinensis* 3 months** | 5 | 125.06 ± 7.894 | 8.142 ± 0.66*** | 0.065 ± 0.006***^###@@@^ | 1.131 ± 0.105 | 0.09 ± 0.001 |

*compared to the uninfected group, ^#^compared to the *O. viverrini* -infected group, ^$^compared to the *C. sinensis* -infected group, ^@^compared to the *O. felineus* -infected group. *, ^#^, or ^$^: P < 0.05; **, ^##^, or ^$$^: P < 0.01; ***, ^###^, or ^$$$^: P < 0.001;

P values were obtained by the ANOVA + *post hoc* Tukey’s test, mean ± SD (STATISTICA 6.0.).
