# Supplementary material for: Species-specific renal and liver responses during infection with food-borne trematodes Opisthorchis felineus, Opisthorchis viverrini, or Clonorchis sinensis
Source: PLoS One. 2024 Dec 5;19(12):e0311481. doi: 10.1371/journal.pone.0311481 (PMC11620611; doi:10.1371/journal.pone.0311481)
Supplement: S3 Table — The values used to build heatmap at Fig 2B. (DOCX) [file pone.0311481.s004.docx]

**Supplementary Table 3. The results of the** **liver semiquantitative histological analysis of animals infected with *O.felineus*, *O.viverrini* or *C. sinensis* at 1 and 3 months post infection.**

|  | 1 month | | | | 3 months | | |
| --- | --- | --- | --- | --- | --- | --- | --- |
|  | Uninfected  Mean ± SD | *O.felineus*  Mean ± SD | *O.viverrini*  Mean ± SD | *C.sinensis*  Mean ± SD | *O.felineus*  Mean ± SD | *O.viverrini*  Mean ± SD | *C.sinensis*  Mean ± SD |
| Inflammation | 0.057±0.12 | **13.65 ± 2.2 * #** | **8.09 ± 2.5 *** | **20.7 ± 3 *#** | **15.22 ± 4.1 *** | **14.4 ± 4.9 *** | **16.2 ± 4.5 *** |
| Periductal fibrosis | 0 | **34.79 ± 5.32 *#** | **15.77 ± 3.7 *** | **40.15 ± 13.6 *#** | **47.2 ± 11.3 *#** | **29.43 ± 1.4 *** | **40.56 ± 7.4*#** |
| Bile duct proliferation | 0 | **10.04 ± 4.5 *#** | 0.51 ± 1.1 | **14.85 ± 4.4 *#** | **22.87 ± 2.9 *#** | **9.04 ± 1.5 *** | **28.26 ± 13.7 *#** |
| Epithelium hyperplasia | 0 | **4.59 ± 1.3 *#** | **7.22 ± 1.96 *** | **3.99 ± 1.7 *#** | **5.48 ± 1.5 *#** | **9.96 ±3.4 *** | **4.5 ± 0.9 *#** |
| Biliary neoplasia | 0 | 0.35 ± 0.4 | 0.06 ± 0.09 | 0.38 ± 0.4 | **2.64 ± 1.5 *** | **4.66 ± 2 *** | **1.83 ± 1.2 *** |

For histopathological analysis, the tissue slides were stained with hematoxylin and eosin or Masson’s Trichrome dye by the standard methods and examined under a light microscope (Axioskop 2 Plus; Zeiss, Germany). Histological features of liver were manually scored by two independent investigators and a senior pathologist confirmed the score. A scoring method of type Ratio (morphometry) of data measurements was applied (Gibson-Corley, 2013). This method is based on counting several fields of tissue (e.g. ten random x 400 fields) for each animal, each field scored and a mean score assigned for the whole tissue of that animal. Each slice of a lobe was analyzed in all fields of view (20–30 fields). Each field of view was divided into 100 squares. Inflammatory cell infiltration, cholangiocyte hyperplasia, Biliary neoplasia, periductal fibrosis, and bile duct cell proliferation were assessed by means of a percentage of the area (the number of squares occupied).

P values were obtained by the Mann–Whitney U test. * - compared to the uninfected group, #—compared to the OV-infected group; *# p< 0.05; **## p< 0.01.

SD - standard deviation.
