# Supplementary material for: Species-specific renal and liver responses during infection with food-borne trematodes Opisthorchis felineus, Opisthorchis viverrini, or Clonorchis sinensis
Source: PLoS One. 2024 Dec 5;19(12):e0311481. doi: 10.1371/journal.pone.0311481 (PMC11620611; doi:10.1371/journal.pone.0311481)
Supplement: S4 Table — (DOCX) [file pone.0311481.s005.docx]

**Supplementary Table 4. Serum and urine biochemistry of animals infected with *O.felineus*, *O.viverrini* or *C. sinensis* at 1 and 3 months post infection.**

| Biochemical parameters | 1 month | | | | 3 months | | |
| --- | --- | --- | --- | --- | --- | --- | --- |
|  | Uninfected  Mean ± SD | *O.felineus*  Mean ± SD | *O.viverrini*  Mean ± SD | *C.sinensis*  Mean ± SD | *O.felineus*  Mean ± SD | *O.viverrini*  Mean ± SD | *C.sinensis*  Mean ± SD |
| ALT (U/L) | 32 ± 8.1 | 84.69 ± 30.9 ** | 107.1 ± 20.5  ** | 51.7 ± 28.2 | 115 ± 30.1 * $$ | 137.8 ± 46.4 ** $$ | 27.1 ±3.7 |
| AST (U/L) | 32.3 ± 24.3 | 40.3 ± 13.1 | 81.1 ± 34.9 ** | 45.1 ± 14.8 | 45.6 ± 15.5 | 55.91 ± 22.33 | 44.5 ± 20.5 |
| Total cholesterol, (mmol/L) | 2.7 ± 0.8 | 3.7 ± 1.1 | 2.9 ± 0.4 | 3 ± 0.6 | 3.4 ± 0.7 | 3.3 ± 0.4 | 2.8 ±0.4 |
| Triglycerides, (mmol/L) | 3.7 ± 2.28 | 3.82 ± 1.74 | 5.4 ± 1.1 * | 7.1 ± 1.8 * | 2.6 ± 1.026 | 7.15 ± 1 * | 6 ± 1.2 * |
| Serum Il-6 (pg/mL) | 67.9 ± 24.4 | 233.5 ± 161.8 | 257.4 ± 71 * | 113.6 ± 35 | 269.6 ± 174.3 | 129.6 ± 125.4 | 183.5 ± 178 |
| Serum Il-17 (pg/mL) | 83.5 ± 17 | 91.1 ± 9.6 | 135.5 ± 70.4 | 77.5 ± 54 | 86.3 ± 39 | 108.3 ± 14.4 | 82 ± 33.3 |
| Serum TNFα (pg/mL) | 19.3 ± 6.6 | 120.2 ± 66 | 143.1 ± 75 * | 61.5 ± 30 | 118.8 ± 28 * | 104.5 ± 92.7 | 117.5 ± 95.4 |
| Urine protein  (mg/L) | 1±0.4 | 0.96±0.09 | 0.94±0.7 | 1.54±0.46 | 1.4±0.7 ****#** | 0.68±0.33 | 0.95±0.02 |
| Urine creatinine (µmol/L) | 74±27.4 | 385.7±  176.5** | 185.1±  87.7 | 466.2±  221.5** | 128.6 ± 80.2 | 74.1 ± 27.1 | 68.6 ± 37.5 |
| Serum creatinine (µmol/L) | 113±32 | 227.45± 8.3** | 220± 25.3** | 130± 28.3 | 237± 39.1 ** | 226.7 ± 58.5 ** | 204± 51.2* |
| Serum KIM-1 (mg/L) | 27.5±26 | 135.7±75.3* | 116.2±24.5 | 87.9±42.34 | 39.5±25.1 | 50.5±36.6 | 69.5±11.9 |

P values were obtained by the Mann–Whitney U test. * - compared to the uninfected group, # -compared to the *O.viverrini* -infected group; $ - compared to the *C.sinensis* -infected group *#$ p< 0.05; **##$$ p< 0.01. parameter values taken from (Van Hoosier, G.L., McPherson, C.W., 1987. Laboratory hamster. Academic Press Inc., New York) Abbreviation: SD - standard deviation; ALT - Alanine transaminase; AST - Aspartate aminotransferase; IL-6 – Interleukin 6; IL-17 – Interleukin 17; TNFa – Tumor necrosis factor alpha; KIM-1 – Kidney injury molecule 1.
