# Supplementary material for: Species-specific renal and liver responses during infection with food-borne trematodes Opisthorchis felineus, Opisthorchis viverrini, or Clonorchis sinensis
Source: PLoS One. 2024 Dec 5;19(12):e0311481. doi: 10.1371/journal.pone.0311481 (PMC11620611; doi:10.1371/journal.pone.0311481)
Supplement: S5 Table — The values used to build heatmap at Fig 5B. (DOCX) [file pone.0311481.s006.docx]

**Supplementary Table 5. The results of the** **kidney semiquantitative histological analysis of animals infected with *O.felineus*, *O.viverrini* or *C. sinensis* at 1 and 3 months post infection (mean ± SD).**

| Group | Bowman’s space area  Mean ± SD | Tubular casts in cortex  Mean ± SD | Tubular casts in medulla  Mean ± SD | Mesangial matrix  Mean ± SD | Interstitial fibrosis  Mean ± SD |
| --- | --- | --- | --- | --- | --- |
| Uninfected | 0.061 ± 0.035% | 0.005 ± 0.008% | 0.016 ± 0.029% | 21.34 ± 4% | 0.02 ± 0.03% |
| *O. felineus*  1 month | 0.094 ± 0.036% | 0.047 ± 0.066% | 0.247 ± 0.339 % | 30.28 ± 2.3 % | **0.43 ± 0.22%*#** |
| *O. viverrini*  1 month | 0.058 ± 0.033% | 0.131 ± 0.133% | 0.159 ± 0. 246 % | 26.91 ± 2.15 % | 0.04 ± 0.04 % |
| *C. sinensis*  1 month | **0.11 ± 0.04% *** | 0.01 ± 0.013% | 0.069 ± 0.112% | 24.33 ± 4.53 % | **0.51 ± 0.18 %*#** |
| *O. felineus*  3 months | **0.130 ± 0.041% *** | 0.012 ± 0.019% | 0.113 ± 0.135% | **27.22 ± 3.96 % *** | **0.53 ± 0.28% *#** |
| *O. viverrini*  3 months | **0.121 ± 0.042% *** | 0.023 ± 0.022% | 0.000 ± 0.000% | **30.96 ± 4.4 % **** | 0.065 ± 0.09% |
| *C. sinensis*  3 months | **0.106 ± 0.048% *** | 0.027 ± 0.023% | 0.040 ± 0.066% | 24.82 ±30,11/,66 % | 0.35 ± 0.24% |

P values were obtained by the Mann–Whitney U test. * - compared to the uninfected group, #—compared to the *O. viverrini* -infected group; *# p< 0.05; **## p< 0.01.

To assess structural kidney changes, 20 random fields of view (x200 magnification) were selected. Each field of view was subdivided into 100 equal square units (conditional units) in the ImageJ software. The area of the casts is presented as a percentage of the number of squares occupied. The percentage of the area occupied by the mesangial matrix was calculated from the area of each glomerulus. For each animal, 10 glomeruli were randomly chosen. Bowman’s space area was estimated according to the following algorithm: each capsule space area was assigned a value from zero to 1.0, where zero is the absence of expansion, and 1.0 denotes capsule expansion by more than 15 μm. Each field of view was scored, and a mean score was assigned to the whole tissue of that animal (Klopfleisch, 2013).
